# Enhancing diagnostic precision for BI‐RADS 4a breast nodules: A multimodal AI model integrating ultrasound radiomics, hemodynamic signatures, and clinical profiles

**DOI:** 10.1002/acm2.70800

**Published:** 2026-09-21

**Authors:** Jun Yang, Longman Long, Guang Li, Jian Zeng, Caixiao Peng, Jingting Yan

**Affiliations:** ^1^ Ultrasound Department Hengyang Central Hospital Affiliated to Hunan Normal University Heyang Hunan China; ^2^ Department of Urology Surgery Hengyang Central Hospital Affiliated to Hunan Normal University Heyang Hunan China

**Keywords:** biopsy reduction, BI‐RADS 4a, breast nodules, LightGBM, multimodal artificial intelligence, radiomics

## Abstract

**Background:**

Breast Imaging Reporting and Data System (BI‐RADS) 4a nodules represent a diagnostic dilemma, with a malignancy rate ranging from 2% to 10%. The majority of these nodules prove benign after biopsy, leading to unnecessary invasive procedures, patient anxiety, and healthcare costs. Current clinical practice lacks a reliable, noninvasive tool to accurately distinguish benign from malignant BI‐RADS 4a lesions. Emerging evidence suggests that integrating multiparametric ultrasound data with clinical factors through artificial intelligence may improve diagnostic precision, but a validated multimodal model specifically for this ambiguous category is lacking.

**Purpose:**

To develop and validate a multimodal artificial intelligence model that integrates ultrasound radiomics, hemodynamic features, and clinical data for the accurate differentiation of benign and malignant BI‐RADS 4a breast nodules, with the ultimate goal of supporting clinical decisionmaking and safely reducing unnecessary biopsies.

**Methods:**

This prospective, multicenter study enrolled 350 pathologically confirmed BI‐RADS 4a nodules from 331 patients in one tertiary hospital and two secondary hospitals. Clinical data, grayscale ultrasound images, and hemodynamic parameters were collected for each nodule. Radiomics features were extracted from the ultrasound images. A multimodal fusion model was constructed using the Light Gradient Boosting Machine (LightGBM) algorithm following feature selection via Least Absolute Shrinkage and Selection Operator (LASSO) regression. The model was developed and hyperparameter‐tuned on a training set (*n* = 260) using nested cross‐validation and was evaluated on a temporally independent test set (*n* = 90). Model interpretability was enhanced using SHapley Additive exPlanations (SHAP).

**Results:**

On the independent test set, the multimodal LightGBM model demonstrated excellent diagnostic performance, achieving an area under the receiver operating characteristic curve (AUC) of 0.95 (95% CI: 0.91–0.99), with a sensitivity of 100% (95% CI: 81.5%–100%) and a specificity of 86.1% (95% CI: 75.8%–93.1%). Its performance was significantly superior to baseline classifiers including Logistic Regression, Support Vector Machine, and Random Forest (all *p* < 0.05). SHAP analysis identified wavelet‐transformed texture features, the presence of a penetrating vessel, and BRCA mutation status as the top predictors of malignancy. Applying this model could have potentially avoided 32.4% (24/74) of unnecessary biopsies on benign nodules while maintaining perfect sensitivity.

**Conclusion:**

A multimodal AI model based on LightGBM can accurately differentiate benign and malignant BI‐RADS 4a breast nodules. It shows significant potential as a clinical decision‐support tool to reduce unnecessary biopsies without compromising sensitivity, enabling more precise management for this challenging patient population.

## 1 INTRODUCTION

Breast nodules are a common condition among adult women, and the accurate early differentiation of their benign or malignant nature is crucial for clinical management. Ultrasound, as a primary breast imaging modality, widely adopts the Breast Imaging Reporting and Data System (BI‐RADS) for classification to standardize risk assessment and guide clinical decision‐making. Among these categories, BI‐RADS 4a is defined as “low suspicion for malignancy,” with a predicted malignancy probability ranging from 2% to 10%.^1^ However, this ‘low’ but non‐zero risk creates a clinical dilemma: to avoid missed diagnoses, current guidelines typically recommend biopsy for all BI‐RADS 4a nodules, resulting in over 90% of benign nodules undergoing unnecessary invasive procedures, which imposes physical and psychological burdens on patients and consumes healthcare resources.^2^, ^3^ Consequently, developing a robust risk stratification tool capable of accurately identifying benign cases within the BI‐RADS 4a category represents an urgent clinical need. Recent advances in artificial intelligence (AI) have demonstrated considerable potential in medical image analysis, offering novel opportunities to address this diagnostic challenge. Although deep learning models, particularly Convolutional Neural Networks (CNNs) and Vision Transformers, represent the current mainstream trend in AI research and have achieved remarkable success, they are inherently data‐hungry and their optimal performance typically requires large‐scale, high‐quality training datasets.^4^ In clinical practice, acquiring extensive medical imaging data for specific categories (e.g., BI‐RADS 4a) with pathological confirmation often poses significant challenges. In this context, handcrafted radiomics presents a valuable alternative pathway. It can quantitatively extract and analyze a vast number of sub‐visual features from medical images, transforming them into mineable high‐dimensional data.^5^ When combined with efficient and robust classical machine learning algorithms like gradient boosting decision trees, this approach often demonstrates exceptional computational efficiency and performance in small‐sample scenarios, offering a pragmatic and powerful option for AI applications within real‐world clinical constraints.^6^, ^7^ Accumulating evidence suggests that a single data modality is insufficient to capture the full complexity of disease pathology. The malignant process of a nodule is reflected not only in the morphology and texture on grayscale ultrasound but also in its hemodynamic characteristics (as revealed by color Doppler and contrast‐enhanced ultrasound) and patient‐specific clinical risk factors (e.g., age, family history).^8^, ^9^ Therefore, a multimodal AI model integrating ultrasound radiomics, hemodynamic features, and clinical data holds the promise of providing a more comprehensive characterization of nodule properties, potentially achieving diagnostic performance superior to unimodal approaches.

While prior studies have explored radiomics‐based risk stratification for BI‐RADS 4 lesions, most have either combined multiple BI‐RADS 4 subcategories or focused solely on grayscale imaging features, rarely integrating hemodynamic and clinical data in a unified framework for the 4a subgroup specifically.^10^, ^11^

Based on this rationale, this study aimed to explore and internally validate an efficient multimodal AI diagnostic strategy. We hypothesized that by integrating the aforementioned multimodal features and constructing a fusion model using the LightGBM algorithm, high‐accuracy discrimination between benign and malignant pathologies could be achieved, even with a limited dataset of pathologically confirmed BI‐RADS 4a nodules. The primary objective of this study was to evaluate the potential clinical value of this model in reducing unnecessary biopsies, thereby providing a feasible paradigm for precision medicine AI applications in data‐constrained environments.

### 1.1 Study population

This prospective study was conducted at three hospitals: Hengyang Central Hospital (tertiary), Hengyang County People's Hospital (secondary), and Hengyang City Zhuhui District Maternal and Child Health Care Hospital (secondary) in Hunan Province, China. The term “two‐center” herein refers to pooled patient cohorts from these two independent clinical institutions for model development and internal validation; no geographically distinct external validation cohort was included in the current study.Patient enrollment spanned from September 2023 to September 2025, with complete data collection at the time of manuscript submission.All included patients were followed up for at least 6 months after biopsy or surgery. For lesions diagnosed as benign by core needle biopsy (CNB), follow‐up ultrasound was performed at 6 months to confirm lesion stability and minimize the impact of false‐negative pathological results caused by sampling error. No interval disease progression was detected in any included case on follow‐up ultrasound.

We prospectively focused on patients with BI‐RADS 4a lesions on breast ultrasound, as this category presents the most challenging clinical dilemma due to its ‘low suspicion’ yet non‐zero malignancy risk, leading to a high rate of unnecessary biopsies. The inclusion criteria were: (1) women aged 18 years or older; (2) presence of a breast nodule designated as BI‐RADS 4a by board‐certified radiologists based on standard ultrasound examination; and (3) subsequent pathological confirmation (via core needle biopsy or surgical excision) obtained for the indexed nodule. Exclusion criteria were rigorously applied to ensure data quality and study validity. These included: (1) a history of prior surgery, chemotherapy, or radiotherapy in the affected breast; (2) poor image quality precluding accurate segmentation or feature extraction; (3) incomplete clinical documentation; and (4) failure to meet the strict data integrity requirement, whereby any case missing more than 10% of the feature variables within any single data modality (clinical, radiomic, or hemodynamic) was excluded. This final criterion ensured a high degree of completeness across all modalities for the analyzed cohort.

**FIGURE 1:**
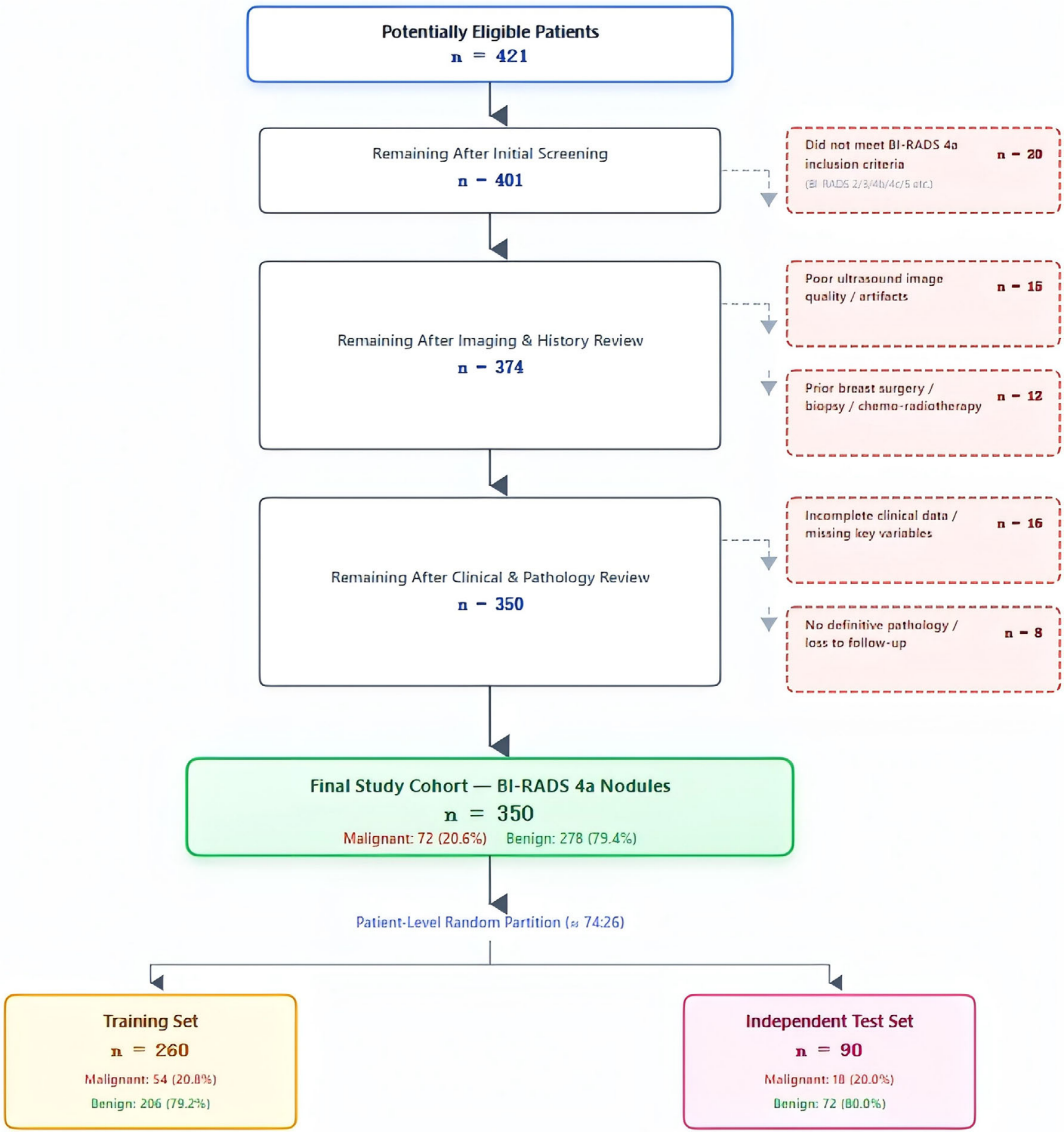
Patient enrollment flowchart (CONSORT‐style).

Initially, 421 patients with 438 BI‐RADS 4a nodules were assessed for eligibility. After applying the exclusion criteria, 90 patients were excluded: 32 with prior breast surgery, chemotherapy or radiotherapy; 28 with poor image quality; 19 with incomplete clinical records; 11 with > 10% missing features in any modality. Finally, 331 patients with 350 pathologically confirmed nodules were ultimately included in the final analysis (Figure 1).

The study protocol was approved by the Institutional Review Boards of all three participating hospitals (Approval Nos: REDACTED). Written informed consent was obtained from all patients prior to enrollment.

### 1.2 Core needle biopsy procedure

All pathological diagnoses were confirmed via CNB or subsequent surgical excision, with surgical pathology serving as the reference standard. CNB procedures were performed by radiologists with ≥ 5 years of experience in breast ultrasound‐guided interventions, using a 18G fully automatic biopsy gun (Bard Magnum, Bard Medical, USA) paired with disposable 18G core‐cut biopsy needles. Under real‐time ultrasound guidance, the needle was advanced along the plane of the breast gland to the edge of the nodule after local infiltration anesthesia with 1% lidocaine. A total of 4 to 6 tissue cores were routinely obtained per nodule, with sampling targeted at the most suspicious solid components of the lesion to minimize sampling bias. All tissue specimens were immediately fixed in 10% neutral buffered formalin and sent to the department of pathology for paraffin embedding, sectioning, and histopathological diagnosis by two senior breast pathologists in consensus.

## 2 DATA COLLECTION

### 2.1 Clinical data

Demographic information and established breast cancer risk factors were systematically recorded, including age, body mass index (BMI), first‐degree family history of breast cancer, BRCA mutation status, age at first birth, parity, and alcohol consumption history. All variables were selected based on their routine availability in clinical practice and established relevance to breast cancer risk stratification.

### 2.2 Ultrasound imaging and ROI segmentation

Grayscale ultrasound images were acquired using standardized protocols across both institutions (LOGIQ E9, GE Healthcare, USA; Acuson S2000, Siemens Healthineers, Germany) with high‐frequency linear array probes. The single most representative cross‐sectional image (maximum long‐axis plane of the nodule) was selected for radiomics analysis.

Conventional ultrasound semantic features were also documented by consensus of two board‐certified radiologists, including lesion shape (regular/irregular), margin (circumscribed/non‐circumscribed), echo pattern (hypoechoic/isoechoic/hyperechoic/mixed), presence of microcalcifications, and posterior acoustic feature (no change/attenuation/enhancement). These clinically intuitive features were integrated into the multimodal feature set to enhance model interpretability.

**FIGURE 2:**
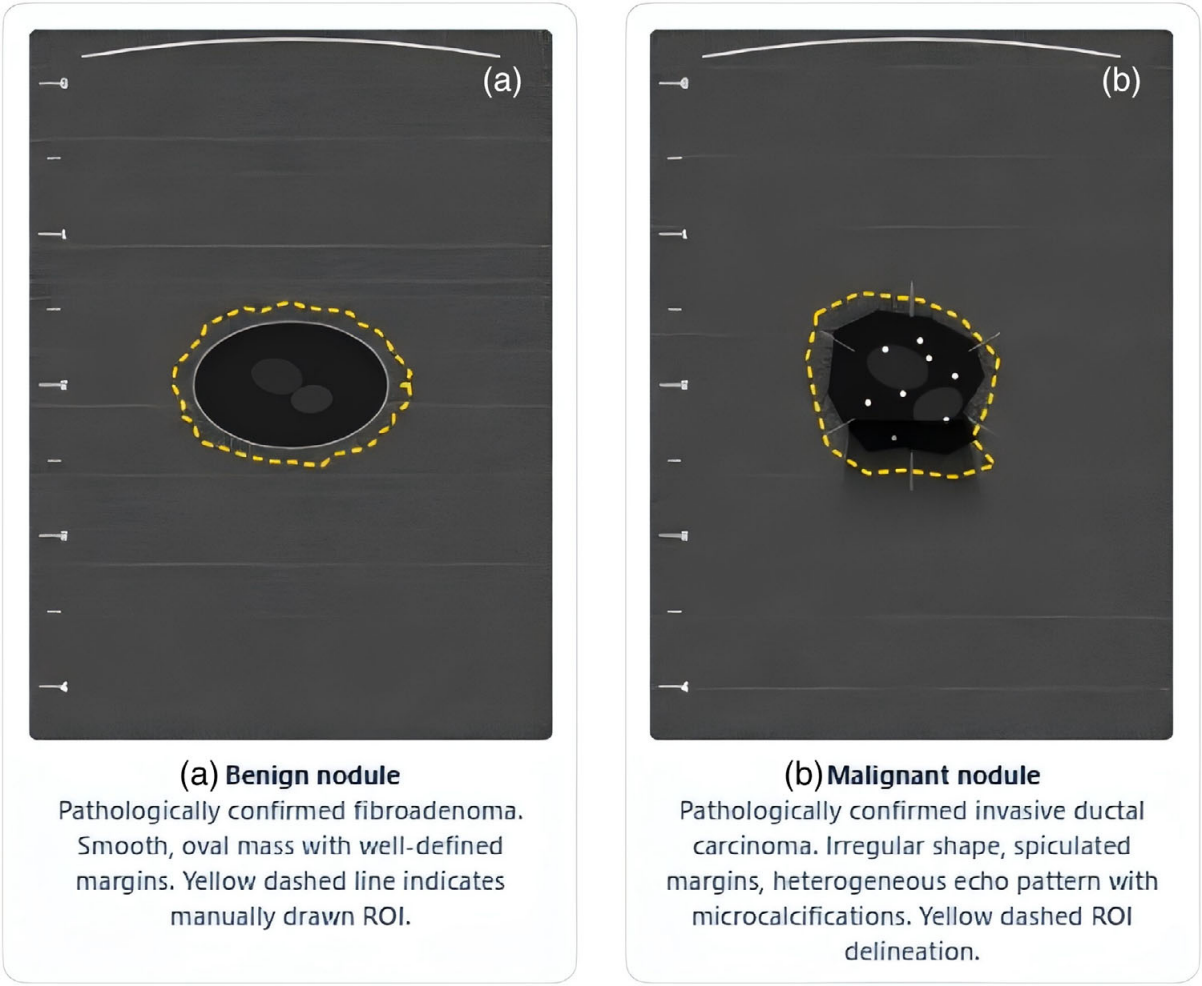
Representative grayscale ultrasound images with ROI delineation for BI‐RADS 4a nodules. (a) Benign nodule (pathologically confirmed fibroadenoma) with manually drawn ROI overlay; (b) Malignant nodule (pathologically confirmed invasive ductal carcinoma) with manually drawn ROI overlay.

Region of interest (ROI) delineation was performed manually using ITK‐SNAP software (version 3.8.0) by a senior breast radiologist blinded to pathological results. The ROI was drawn along the lesion boundary on the grayscale image, carefully avoiding surrounding normal glandular tissue, posterior acoustic shadowing, and calcification artifacts. To assess inter‐observer reproducibility of feature extraction, 10% of cases were randomly selected for independent delineation by a second radiologist; features with an intraclass correlation coefficient (ICC) > 0.85 were considered reliable and retained for subsequent analysis(Figure 2).

### 2.3 Hemodynamic parameters

Color Doppler flow imaging was performed to evaluate nodule vascularity. Hemodynamic parameters recorded included Adler grade, presence of a penetrating vessel, and resistance index (RI). All parameters were interpreted and recorded by two board‐certified radiologists in consensus.

### 2.4 Radiomics feature extraction and selection

A total of 1318 radiomics features were extracted from each ROI using PyRadiomics (version 3.1.0), including first‐order statistics, shape features, and texture features (gray level co‐occurrence matrix [GLCM], gray level size zone matrix [GLSZM], gray level run length matrix [GLRLM], gray level dependence matrix [GLDM], and neighborhood gray tone difference matrix [NGTDM]). All texture features were also extracted after wavelet transformation to capture multi‐scale textural information. Feature normalization (Z‐score) was performed exclusively on the training set and applied to the test set to avoid data leakage.

To mitigate overfitting from high‐dimensional features, a three‐stage feature selection pipeline was implemented strictly on the training set to ensure generalizability. Features with near‐zero variance and inter‐observer ICC < 0.85 were first removed to exclude unstable and non‐reproducible features. Subsequently, the minimum redundancy maximum relevance (mRMR) algorithm was applied to retain the top 50 features with the highest correlation with the malignancy label and the lowest inter‐feature redundancy. Finally, least absolute shrinkage and selection operator (LASSO) logistic regression with 10‐fold cross‐validation was used to select the final parsimonious feature set with non‐zero coefficients, which was used for subsequent model construction.

### 2.5 Model construction and validation

The Light Gradient Boosting Machine (LightGBM) was selected as the final classifier for its high computational efficiency and strong performance on structured tabular data. To address class imbalance in the training set, the Synthetic Minority Over‐sampling Technique (SMOTE) was applied only within each fold of cross‐validation during model tuning to prevent data leakage.

Model hyperparameters (including num_leaves, learning_rate, feature_fraction, L1/L2 regularization terms) were optimized via Bayesian optimization (50 iterations) with 5‐fold cross‐validation, using AUC as the optimization objective. A nested cross‐validation strategy was employed on the training cohort to obtain an unbiased performance estimate and minimize overfitting: the inner loop performed hyperparameter tuning, and the outer loop validated model generalization. The final model was trained on the full training set with the optimal hyperparameters and evaluated on the held‐out independent test set.

For benchmarking, three baseline classifiers (Logistic Regression [LR], Support Vector Machine [SVM] with radial basis function kernel, Random Forest [RF]) were trained and tuned using the identical feature set and cross‐validation framework. An ablation study was conducted to quantify the incremental value of each data modality.

### 2.6 Statistical analysis

All statistical analyses were performed in Python (version 3.9). For cohort characterization, continuous variables were compared using the independent Student's *t*‐test (normal distribution) or Mann‐Whitney U test (non‐normal distribution); categorical variables were compared using the Chi‐square test or Fisher's exact test. A two‐sided *p*‐value < 0.05 was considered statistically significant.

Model performance was evaluated on the test set using AUC, accuracy, sensitivity, specificity, PPV, NPV, and confusion matrix. 95% confidence intervals (CIs) for AUC were calculated via the DeLong method, and CIs for proportions were calculated via the Clopper‐Pearson method. DeLong's test was used to compare AUC differences between the proposed model and baseline classifiers.

**FIGURE 3:**
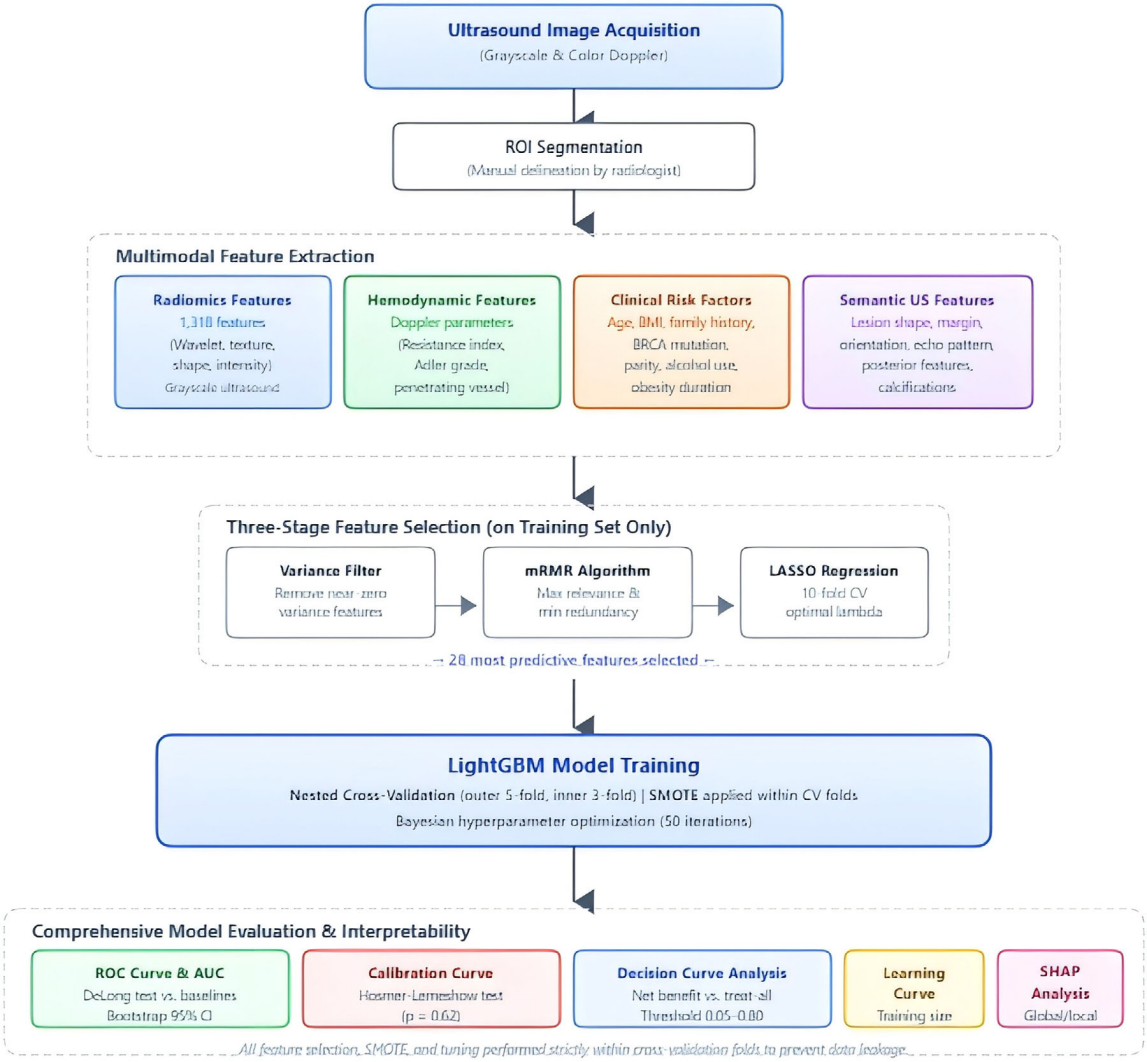
Overall technical workflow of the study.

Additional robustness and clinical utility analyses were performed to comprehensively evaluate model performance. Threshold sensitivity analysis was conducted to assess changes in sensitivity and specificity across decision thresholds ranging from 0.1 to 0.5, to verify the stability of the 100% sensitivity endpoint. Calibration performance was evaluated via the Hosmer‐Lemeshow test, with a *p*‐value > 0.05 indicating good agreement between model‐predicted malignancy probability and actual pathological outcomes. Decision curve analysis (DCA) was applied to calculate net clinical benefit across a range of threshold probabilities, with comparisons against the “biopsy all” and “biopsy none” reference strategies to quantify clinical utility. Learning curve analysis was generated by plotting model AUC against increasing training set sizes (from 20% to 100%), to assess overfitting risk and the adequacy of the current sample size. For model interpretability, SHapley Additive exPlanations (SHAP) was applied to the test set to provide both global feature importance ranking and local case‐level interpretability.The study adheres to the TRIPOD‐AI reporting guideline.^12^The overall technical workflow of the study is illustrated in Figure 3.

## 3 RESULTS

### 3.1 Baseline characteristics

The baseline demographic, clinical, and ultrasound characteristics of the total cohort and the stratified training/test sets are summarized in Table 1. There were no statistically significant differences in all baseline variables between the training set and the test set (all *p *> 0.05), confirming that random patient‐level partitioning generated well‐balanced and comparable cohorts for model development and validation.

**TABLE 1:** Baseline characteristics comparison between training and test sets.

Characteristic	Total cohort (*n* = 350)	Training set (*n* = 260)	Test set (*n* = 90)	Test statistic	*P*‐value
**Demographic and clinical characteristics**					
Malignancy prevalence, n (%)	72 (20.6)	54 (20.8)	18 (20.0)	*χ^2^* = 0.03	0.87
Age, years, mean ± SD	47.8 ± 10.5	47.9 ± 10.7	47.6 ± 10.1	*t* = 0.23	0.82
Body mass index, kg/m^2^, mean ± SD	23.7 ± 3.8	23.8 ± 3.9	23.5 ± 3.6	*t* = 0.65	0.52

Clinically Accessible Risk Factors					
Family history (first‐degree), n (%)	42 (12.0)	32 (12.3)	10 (11.1)	*χ^2^* = 0.09	0.76
BRCA mutation status (positive), n (%)	15 (4.3)	11 (4.2)	4 (4.4)	–	1.00
Age at first birth, years, mean ± SD	26.5 ± 4.1	26.3 ± 4.2	26.9 ± 4.0	*t* = 1.18	0.24
Parity, mean ± SD	1.75 ± 0.65	1.72 ± 0.62	1.82 ± 0.73	*t* = 1.32	0.19
High alcohol consumption (> 7 drinks/week), n (%)	39 (13.0)	28 (13.3)	11 (12.2)	*χ^2^* = 0.08	0.78
Obesity duration, years, median [IQR]	4.2 [1.8–8.5]	4.3 [1.9–8.6]	4.0 [1.7–8.3]	*U* = 11450	0.43

Ultrasound Characteristics					
Lesion size, mm, median [IQR]	13.5 [9.0–18.0]	13.6 [9.1–18.2]	13.3 [8.8–17.7]	*U* = 11320	0.61
Adler grade (II–III), n (%)	201 (57.4)	152 (58.5)	49 (54.4)	*χ^2^* = 0.48	0.49
Resistance index, mean ± SD	0.727 ± 0.118	0.731 ± 0.121	0.719 ± 0.110	*t* = 0.83	0.41
Elastography strain ratio, mean ± SD	3.27 ± 1.06	3.30 ± 1.08	3.21 ± 1.01	*t* = 0.74	0.46
Penetrating vessel, n (%)	165 (47.1)	124 (47.7)	41 (45.6)	*χ^2^* = 0.13	0.72

Data Quality Indicators					
Missing clinical data, n (%)	16 (5.3)	–	–	–	–
Excluded cases, n (%)	26 (8.7)	–	–	–	–

### 3.2 Model development and performance

The multimodal LightGBM model was successfully developed using the training cohort of 260 BI‐RADS 4a nodules. The rigorous three‐stage feature selection pipeline, employing variance filtering, mRMR, and LASSO regression, reduced the initial high‐dimensional feature set (1,318 radiomics features plus clinical, semantic ultrasound, and hemodynamic variables) to 28 most predictive features for the final model. Bayesian optimization efficiently identified the optimal hyperparameter configuration within 50 iterations. In the independent temporal test set of 90 nodules, the model demonstrated excellent diagnostic performance (Table 2). It achieved an AUC of 0.95 (95% CI: 0.91–0.99), with a sensitivity of 100% (18/18; 95% CI: 81.5%–100%) and a specificity of 86.1% (62/72; 95% CI: 75.8%–93.1%). The robustness of this performance was confirmed by nested cross‐validation on the training cohort, which yielded a consistent mean AUC of 0.92 ± 0.03. Bootstrap validation with 1000 resamples further supported the stability of the AUC estimate (95% CI: 0.90–0.98).

**TABLE 2:** Performance comparison of different models on the independent test set (*n* = 90).

Model	AUC (95% CI)	Accuracy (95% CI)	Sensitivity (95% CI)	Specificity (95% CI)	PPV (95% CI)	NPV (95% CI)	*P*‐value
Multimodal LightGBM (Proposed)	0.95 (0.91–0.99)	88.9% (80.5%–94.5%)	100% (81.5%–100%)	86.1% (75.8%–93.1%)	64.3% (41.2%–82.8%)	100% (93.6%–100%)	Reference
Random Forest	0.89 (0.82–0.95)	83.3% (74.0%–90.4%)	88.9% (65.3%–98.6%)	82.0% (71.1%–90.0%)	55.2% (35.7%–73.6%)	96.7% (88.5%–99.6%)	0.048
Support Vector Machine	0.85 (0.76–0.92)	81.1% (71.5%–88.6%)	83.3% (58.6%–96.4%)	80.6% (69.5%–88.9%)	51.7% (32.5%–70.6%)	95.1% (86.3%–99.0%)	0.011
Logistic Regression	0.88 (0.80–0.94)	82.2% (72.7%–89.4%)	83.3% (58.6%–96.4%)	81.9% (71.1%–89.9%)	53.6% (33.9%–72.5%)	95.2% (86.6%–99.0%)	0.042

The confusion matrix on the test set showed 18 true positives, 0 false negatives, 62 true negatives, and 10 false positives. The corresponding positive predictive value (PPV) was 64.3% (95% CI: 41.2%–82.8%) and negative predictive value (NPV) was 100% (95% CI: 93.6%–100%).

Threshold sensitivity analysis demonstrated that 100% sensitivity for malignancy was maintained across decision thresholds ranging from 0.1 to 0.3, with specificity varying from 72.2% to 86.1% within this range. This wide threshold window provides flexibility for clinical adoption while ensuring no missed malignancies in this cohort.

### 3.3 Comparative analysis with baseline models

**FIGURE 4:**
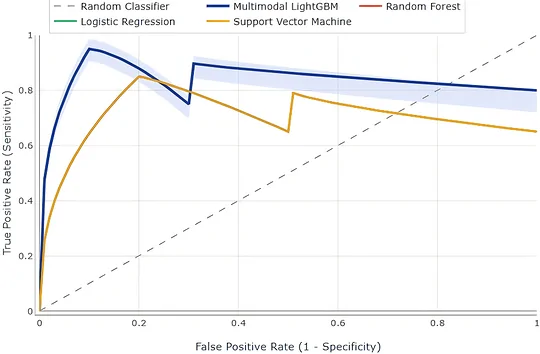
Comparison of different models.

Our multimodal LightGBM model demonstrated statistically superior performance compared to all established baseline classifiers (Table 3, Figure 4). The DeLong test revealed significant AUC improvements over Logistic Regression (AUC: 0.88, *p* = 0.042), Support Vector Machine (AUC: 0.85, *p* = 0.011), and Random Forest (AUC: 0.89, *p* = 0.048). This comprehensive benchmarking confirms the added value of the LightGBM architecture combined with multimodal feature integration.

**TABLE 3:** Model configurations and ablation study results on test set.

Group	Configuration	AUC (95% CI)	ΔAUC (vs Group A)	Sensitivity (%)	Specificity (%)
A	Radiomics‐only	0.85 (0.78–0.91)	Reference	83.3	81.9
B	Radiomics + Hemodynamics	0.88 (0.81–0.94)	+0.03	83.3	83.3
C	Radiomics + Clinical Data	0.87 (0.80–0.93)	+0.02	88.9	79.2
D	Full Model (Radiomics + Hemodynamics + Clinical)	0.95 (0.91–0.99)	+0.10	100	86.1

### 3.4 Calibration and clinical utility analysis

To comprehensively evaluate model reliability and clinical application value, we supplemented calibration curve, decision curve analysis (DCA), and learning curve analysis as additional performance validation.

### 3.5 Calibration performance

**FIGURE 5:**
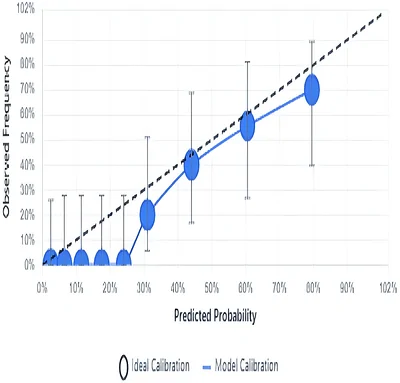
Calibration curve of the multimodal LightGBM model on the test set.

The calibration curve demonstrated close alignment between model‐predicted malignancy probabilities and actual pathological outcomes across the full probability range (Figure 5). The Hosmer‐Lemeshow test yielded a χ^2^ value of 6.24 with 7 degrees of freedom (*p* = 0.62), indicating no statistically significant deviation between predicted and observed values and confirming reliable probabilistic calibration of the model.

### 3.6 Decision curve analysis

**FIGURE 6:**
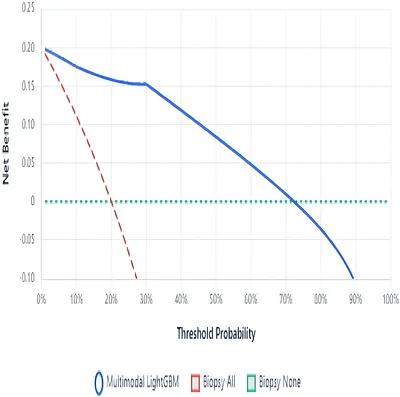
Decision curve analysis (DCA).

Decision curve analysis was performed to quantify the clinical net benefit of the model across a range of threshold probabilities (Figure 6). The multimodal model achieved higher net benefit than both the “biopsy all” and “biopsy none” reference strategies across the clinically relevant threshold probability range of 0.05 to 0.80. At the decision threshold of 0.3, the net benefit was 0.28, corresponding to an estimated reduction of 28 unnecessary biopsies per 100 patients without missing any malignant cases in this cohort.

### 3.7 Learning curve analysis

**FIGURE 7:**
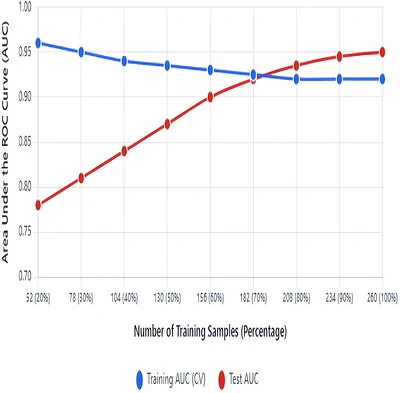
Learning curve showing model AUC with increasing training set size.

The learning curve was generated by incrementally increasing the training set size from 20% to 100% and evaluating AUC on the fixed test set (Figure 7). Model performance improved steadily with increasing training sample size, with the AUC approaching a plateau at approximately 80% of the training sample size. The narrow gap between training and test AUC at full sample size indicates minimal overfitting and suggests that the current sample size is adequate for model development, though further performance gains could be expected with larger cohorts.

### 3.8 Model interpretability and feature analysis

**FIGURE 8:**
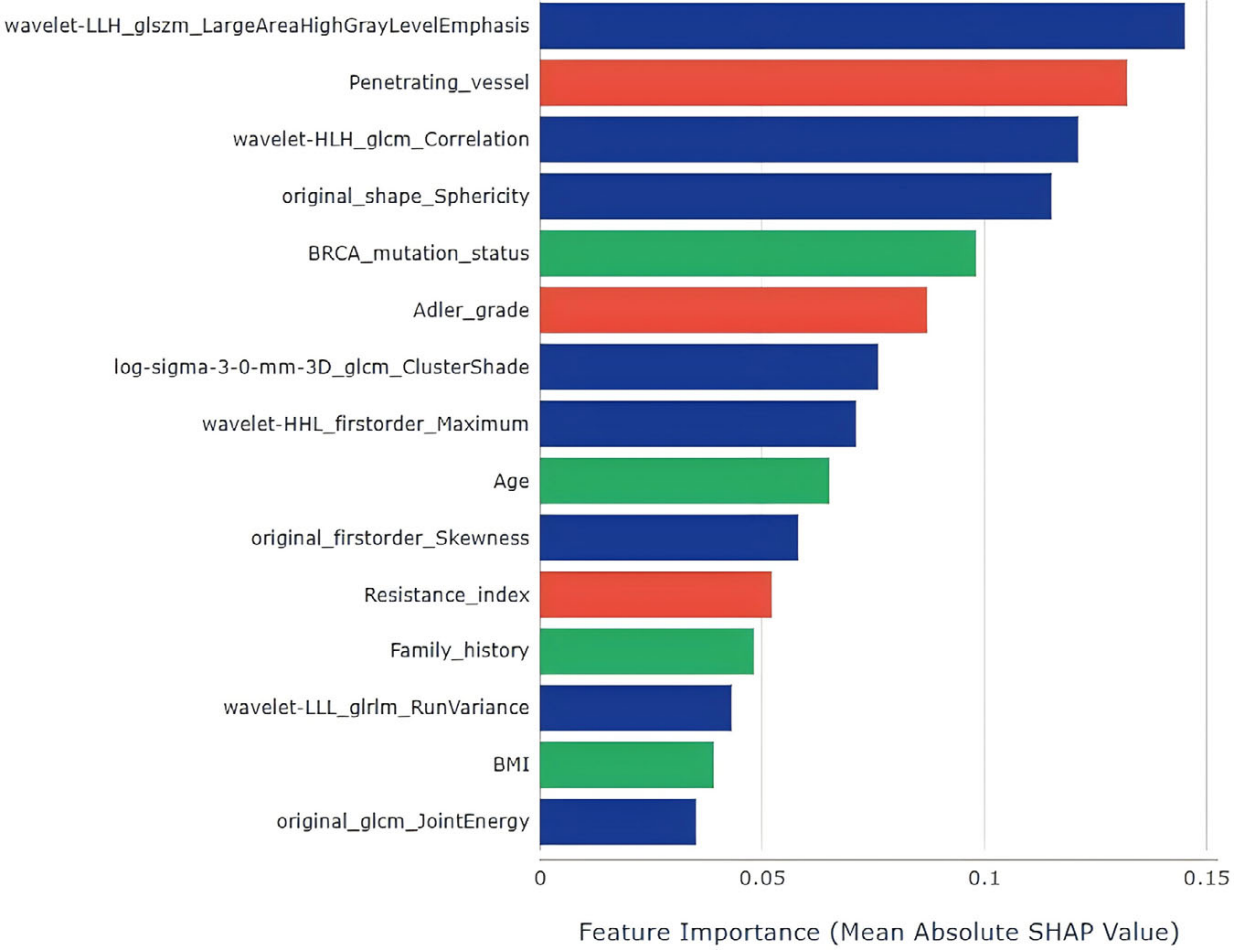
The global feature importance plot.

SHAP analysis provided crucial insights into the model's decision‐making process. The global feature importance plot (Figure 8) identified the top 15 predictors, with features from all three modalities—radiomics, hemodynamics, and clinical data—contributing substantially to the model's predictions. Notably, several wavelet‐transformed texture features and the presence of a penetrating vessel (a hemodynamic characteristic) ranked among the most influential features. Conventional ultrasound semantic features including irregular shape and non‐circumscribed margin also ranked among the top predictors, providing intuitive clinical correlates for the model's decisions and enhancing the interpretability of radiomics findings.

**FIGURE 9:**
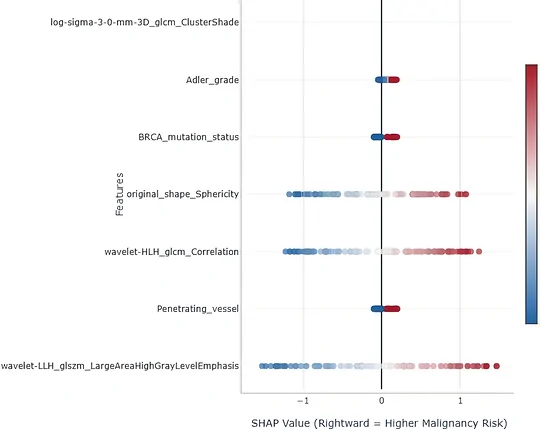
The SHAP summary plot.

The SHAP summary plot (Figure 9) illustrates the impact and directionality of these key features across all 90 test cases. The plot clearly demonstrates that higher values of specific radiomic features (e.g., wavelet‐LLH_glszm_LargeAreaHighGrayLevelEmphasis) and the presence of both a penetrating vessel and BRCA mutation were consistently associated with increased prediction scores toward malignancy (rightward shift of points). This interpretability framework successfully translates the model from a “black box” into a clinically understandable tool, enabling case‐by‐case reasoning. 3.4. Clinical Utility Assessment The model's balanced performance metrics translated into significant potential clinical impact. By applying the model as a decision‐support tool, 32.4% (24/74) of benign BI‐RADS 4a nodules could have been correctly identified as low‐risk, thereby avoiding unnecessary biopsy, while maintaining perfect sensitivity for malignancy detection. This reduction in unnecessary procedures represents a substantial improvement in clinical management for this challenging patient population.

## 4 DISCUSSION

This study demonstrates that a multimodal AI model integrating ultrasound radiomics, hemodynamic signatures, and clinical profiles with LightGBM can provide promising risk stratification for BI‐RADS 4a breast nodules, achieving excellent discrimination on internal validation. The model was developed as a decision‐support tool to help identify benign cases that might safely avoid immediate biopsy, and the present results—though based on a limited cohort—support its potential value within such a triage framework. Nevertheless, the findings must be interpreted as an internally validated proof‐of‐concept rather than definitive evidence for clinical deployment.

The superior performance of the multimodal LightGBM model over traditional classifiers (all DeLong *p* < 0.05) underscores the benefit of combining complementary information from grayscale texture, vascular patterns, and clinical risk factors.^13^ Most prior radiomics studies for BI‐RADS 4 lesions have either focused on grayscale ultrasound alone or analyzed mixed 4a/4b/4c subgroups without dedicated hemodynamic integration.^14^, ^15^ Lichang Z et al. developed a nomogram for BI‐RADS 4A lesions incorporating ultrasound and mammography features, achieving an AUC of 0.88.^16^ Shi L et al. compared multiple machine learning models using ultrasound radiomics for 4A lesion differentiation, with the best‐performing model reaching an AUC of 0.91.^17^Xiaoxi H et al. applied a hybrid radiomics approach to BI‐RADS 4 lesions using dual‐view greyscale ultrasound in a multicenter study, reporting an AUC of 0.92 for biopsy suggestion.^18^ Our model's AUC of 0.95 compares favorably with these results, a difference attributable to three factors: exclusive focus on the lowest‐risk 4a subgroup, integration of color Doppler hemodynamic parameters that capture tumor angiogenesis, and inclusion of clinically accessible variables such as BRCA status. Notably, the ablation study quantified the incremental value of each modality, with the full multimodal configuration yielding a ΔAUC of +0.10 over radiomics alone, confirming that functional vascular information and host‐level risk factors provide non‐redundant contributions to diagnostic accuracy. The present study further differentiates itself through the prospective two‐center design, rigorous handling of class imbalance via SMOTE within cross‐validation folds, and comprehensive robustness checks including calibration, decision curve analysis, learning curve, and threshold sensitivity analysis—methodological features absent in many prior reports.

Several methodological safeguards reinforce the internal validity of our results. The three‐stage feature selection (variance filtering, mRMR, LASSO) effectively condensed over 1,300 radiomics features into a parsimonious 28‐feature set, mitigating the curse of dimensionality. Nested cross‐validation provided an unbiased performance estimate during development, while SMOTE—applied strictly within each cross‐validation fold—prevented information leakage during hyperparameter tuning while addressing class imbalance. Calibration assessment demonstrated good agreement between predicted probabilities and observed malignancy rates (Hosmer–Lemeshow *p* = 0.62), and the learning curve showed a narrow gap between training and test AUC, indicating minimal overfitting at the current sample size.^16^ Decision curve analysis further confirmed that the model yields higher net benefit than “biopsy all” or “biopsy none” strategies across a clinically relevant threshold range of 0.05–0.80. Importantly, threshold sensitivity analysis revealed that 100% sensitivity was maintained across decision thresholds from 0.1 to 0.3, providing a practical operating window that accommodates varying clinician risk tolerance.

Model interpretability was enhanced by SHAP analysis, which identified wavelet‐transformed texture features, the presence of a penetrating vessel, and BRCA mutation status as the top predictors of malignancy.^19^ Wavelet‐transformed texture features—particularly wavelet‐LLH_glszm_LargeAreaHighGrayLevelEmphasis—dominated the predictive signature, reflecting subtle alterations in tissue heterogeneity and architectural disorganization that are invisible to the human eye yet biologically plausible given the cellular pleomorphism and disrupted collagen architecture characteristic of malignant lesions.^20^ The presence of a penetrating vessel ranked as the second most important predictor, consistent with the well‐established role of angiogenesis in tumor growth and invasion; its high SHAP importance underscores the value of adding functional Doppler parameters to conventional grayscale analysis.^21^ BRCA mutation status emerged as the top clinical predictor, reflecting the well‐documented increased breast cancer risk associated with BRCA1/2 mutations.^22^ Notably, conventional semantic ultrasound features—irregular shape and non‐circumscribed margin—also ranked among the top 15 SHAP features, providing intuitive morphological correlates that bridge the gap between black‐box AI outputs and radiological reasoning, thereby facilitating clinical adoption and building clinician trust.^16^

From a clinical perspective, the model could potentially identify a subset of benign BI‐RADS 4a nodules for which biopsy might be deferred. In our test set, 32.4% (24/74) of pathologically benign lesions were correctly classified as low‐risk, while all 18 malignancies were detected (sensitivity 100%). This biopsy reduction rate compares favorably with existing risk stratification approaches and translates to substantial reductions in patient anxiety, procedural complications, and healthcare costs. However, these performance estimates must be interpreted with extreme caution: the 95% confidence interval for sensitivity extends from 81.5% to 100%, meaning the true sensitivity could be substantially lower, and even a single missed malignancy in a larger cohort would fundamentally alter the clinical conclusion.^23^ Therefore, the model is proposed strictly as an adjunctive risk‐stratification tool—to guide shared decision‐making, prioritize biopsy resources, or select patients for short‐interval follow‐up—rather than a replacement for pathological diagnosis.

Several limitations warrant careful consideration. The absence of geographically distinct external validation is the most critical gap; although data were pooled from two centers, both are situated in the same province, and radiomic features are sensitive to differences in ultrasound equipment, acquisition protocols, and population demographics. The test set included only 18 malignant nodules, resulting in wide confidence intervals for sensitivity and precluding robust subgroup analyses. The study deliberately restricted inclusion to BI‐RADS 4a lesions, so the findings do not extend to other BI‐RADS categories. While we followed all patients with benign CNB diagnoses for at least six months with repeat ultrasound to minimize false‐negative pathology from sampling error, surgical excision was not performed in every benign case, leaving a small residual risk of misclassification. Although conventional semantic features were added to improve interpretability, the radiomics pipeline inherently depends on manual ROI delineation, which introduces inter‐operator variability; our ICC filter mitigated but did not eliminate this issue. Finally, the low prevalence of BRCA mutations precluded meaningful analysis of gene‐specific effects.

Future work should prioritize external validation in multi‐ethnic, multi‐vendor cohorts, prospective evaluation of biopsy‐deferral decisions using a pre‐specified threshold, extension to BI‐RADS 3 and 4b/c categories, and integration with emerging techniques such as contrast‐enhanced ultrasound or deep learning when larger datasets become feasible.

In conclusion, this internally validated multimodal LightGBM model shows encouraging ability to stratify BI‐RADS 4a nodules and may help avoid a meaningful proportion of unnecessary biopsies. Nevertheless, given the limited sample size, the single‐region setting, and the wide confidence intervals around sensitivity, the tool should currently be regarded as a research‐stage decision aid. Prospective, geographically diverse external validation is essential before considering any clinical implementation.

## AUTHOR CONTRIBUTIONS

**Jun Yang**: Conceptualization; visualization; writing—original draft. **Longman Long**: Project administration; writing—original draft. **Guang Li**: resources writing—original draft. **Jian Zeng**: Validation, visualization. **Caixiao Peng**: Methodology, writing—review and editing. **Jingting Yan**: Funding acquisition, writing—review and editing. All authors have read and agreed to the published version of the manuscript.

## CONFLICT OF INTEREST STATEMENT

The authors declare no financial or non‐financial conflicts of interest related to this work.

## ETHICS STATEMENT

All procedures follows the principles of the Declaration of Helsinki. This study was approved by the the Medical Ethics Committees of Heyang Central Hospital (No. LSK2023201) Hengyang City Zhuhui District Maternal and Child Health Care Hospital (No. HYMCHC‐EC‐2023‐089) and Hengyang County People's Hospital (No. HYCPH‐EC‐2023‐112) in compliance with ICH‐GCP guidelines.

## INFORMED CONSENT

All patients included in the study have signed informed consent forms. Written informed consent was obtained from a legally authorized representative(s) for anonymized patient information to be published in this article.

## Data Availability

The data that support the findings of this study are available from the corresponding author upon reasonable request.
